# Avoidance of total abdominal wall loss despite torso soft tissue clostridial myonecrosis: a case report

**DOI:** 10.1186/1752-1947-7-5

**Published:** 2013-01-08

**Authors:** Chad Geoffrey Ball, Jean-Francois Ouellet, Ian Bruce Anderson, Andrew Wallace Kirkpatrick

**Affiliations:** 1 Department of Surgery, University of Calgary, Foothills Medical Centre, 1403-29 St. N.W., T2N 2T9, Calgary, AB, Canada

**Keywords:** Biologic prosthesis, Hernia, Necrotizing soft tissue infection

## Abstract

**Introduction:**

Clostridial necrotizing soft tissue infections are often fatal. Myonecrosis of the torso is a particularly lethal combination given the classic need for radical debridement of the abdominal and thoracic walls, and therefore total exposure of the intraperitoneal and intrathoracic viscera. This case is unusual do to our ability to preserve anatomical separation between the viscera and the atmosphere.

**Case presentation:**

We present a 42-year-old Caucasian man with obesity and diabetes who developed clostridial myonecrosis of his right torso following a mesenteric lymph node biopsy. This required an aggressive debridement (sparing subcutaneous flaps and internal oblique aponeurosis) followed by reconstruction of his right hemi-torso with a biologic prosthesis to prevent subsequent hernia formation.

**Conclusion:**

Although basic principles associated with radical debridement were maintained, a full thickness torso wall resection was avoided. This provided reconstruction advantages that included endogenous subcutaneous flap coverage, separation of the peritoneal cavity by the internal oblique aponeurosis, and prevention of a subsequent hernia below the arcuate line. This technique would be of interest to any surgeon or clinician who treats patients with life-threatening torso soft tissue infections.

## Introduction

Necrotizing soft tissue infections comprise a broad spectrum of infectious processes that include, but are not limited to, necrotizing cellulitis, necrotizing fasciitis, and myonecrosis. The classification of these entities is based on the extent of soft tissue involvement as well as the depth of infection [1]. Although the bacteria that cause infections often differ, the general approach to treatment is similar and includes: aggressive resuscitation, broad-spectrum antimicrobial therapy, physiologic support, and immediate radical surgical debridement [2]. The extent of surgical debridement is dictated by the need to achieve margins with normal appearing tissue and vigorous bleeding. This is particularly problematic in cases of trunk myonecrosis because of the requirement for complete abdominal and/or thoracic wall resection, and subsequent exposed intraperitoneal and intrathoracic organs.

## Case presentation

**Figure 1:**
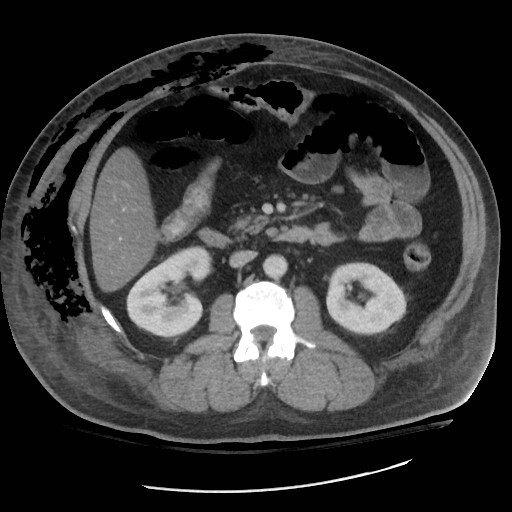
Soft tissue gas of the right abdominal wall.

A 42-year-old, obese, Caucasian male plumber was referred for a mesenteric lymph node biopsy to rule out lymphoma. His medical comorbidities included recent type 2 diabetes, dyslipidemia, and chronic back pain. Physical examination and cross-sectional imaging confirmed abnormal lymphadenopathy limited to the small bowel mesentery. A laparoscopic procedure, converted to 10cm laparotomy, obtained an appropriate nodal excision. The patient began to exhibit increasing oxygen requirements and abdominal discomfort 48 hours after the procedure. His physical examination remained otherwise unremarkable. Computed tomography identified massive right torso soft tissue gas extending from the costal margin to inguinal canal (midline to the back) (Figure 1).

**Figure 2:**
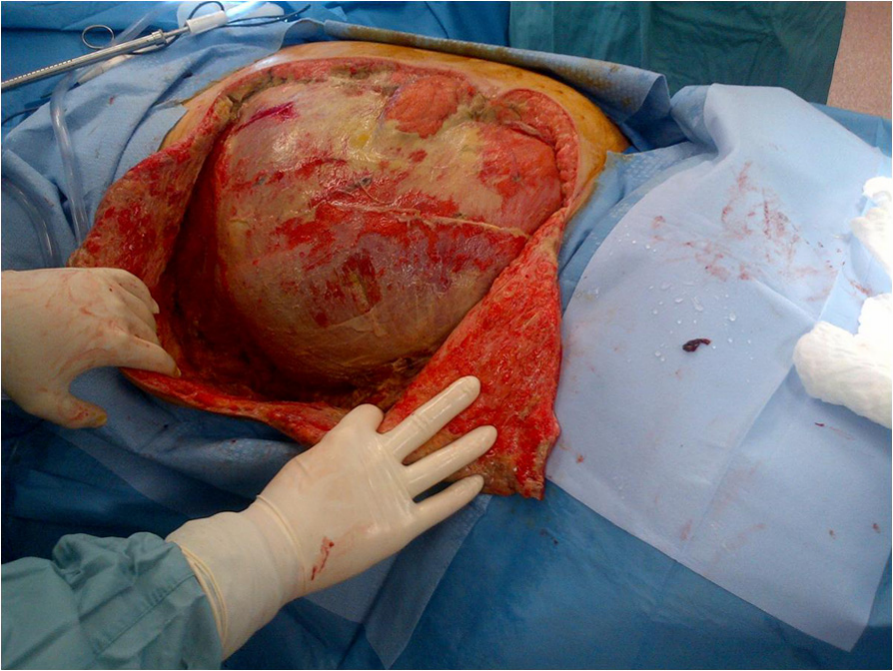
Intact transversus abdominus layer following resection of the external and internal oblique aponeuroses, rectus muscles, and a portion of the pectoralis muscle.

**Figure 3:**
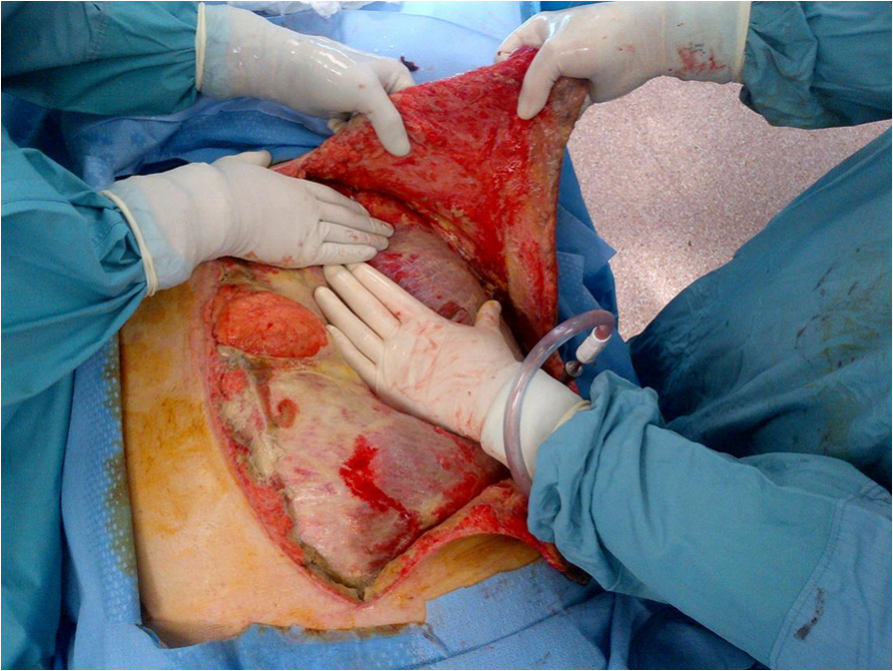
Large triangular skin flaps.

Immediate antimicrobial support and operative intervention led to resection of the entire external oblique and transversus abdominus aponeuroses, rectus muscles, and a portion of the pectoralis muscle of the patient’s right torso (Figure 2). The tissues displayed the classic brown dishwater appearance. The internal oblique aponeurosis and two large triangular skin flaps were left intact (Figure 3). It should be noted that the small laparotomy incision was re-opened to ensure the absence of a missed bowel injury or intraperitoneal source of the contamination. A negative pressure dressing was then applied superficial to the internal oblique layer, followed by loose approximation of the large skin flaps. The patient was returned to the operating room eight hours later because minor additional debridements, washout, and dressing change were required. A total of four subsequent washouts were required. Although he initially suffered significant acute kidney injury, as well as sepsis (white blood cell = 48,000 per cubic millimeter of blood) requiring vasopressor support, these physiologic parameters improved to normal within three days. Final microbiology results confirmed *Clostridium perfringens*. Intravenous immunoglobulin was not required.

**Figure 4:**
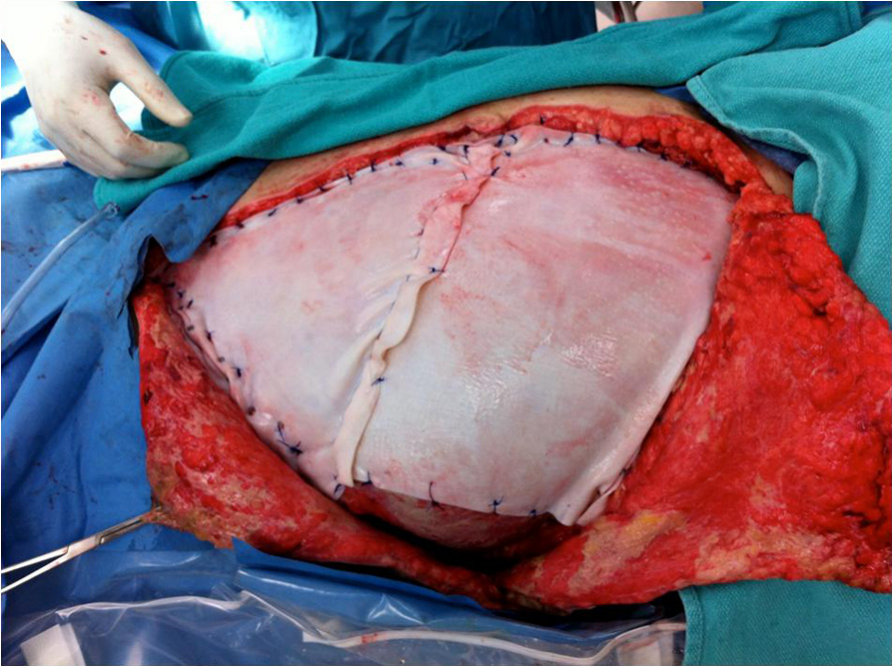
Reconstruction of the abdominal wall with 2 large pieces of biologic graft.

**Figure 5:**
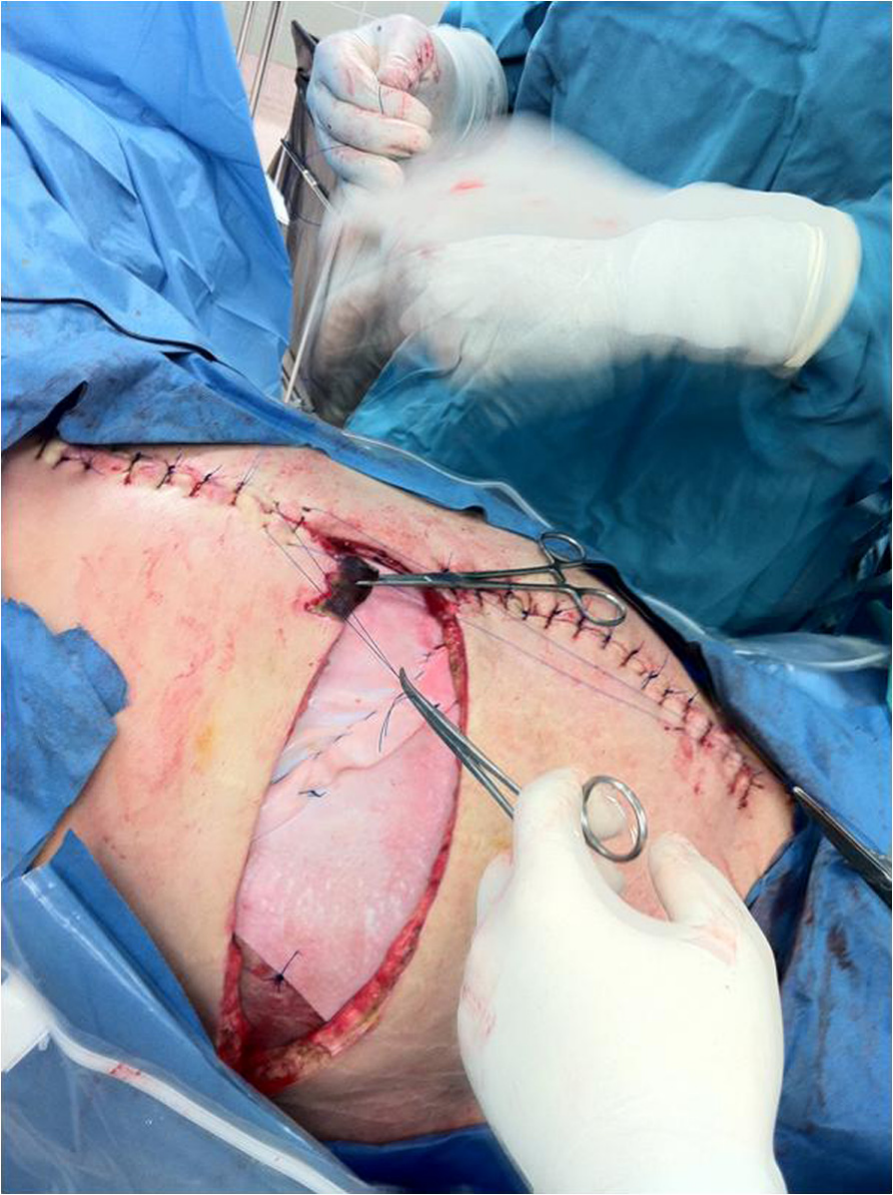
Closure of skin flaps over the abdominal wall reconstruction.

The final closure utilized two large pieces of biologic material (Strattice™, LifeCell Inc., New Jersey, USA) to prevent subsequent hernia formation (Figure 4). This repair was particularly important inferior to the arcuate line, given that only the posterior rectus sheath remained intact throughout his hemi-torso. The biologic material was sewn to the linea alba (medial), costal rib margin (superior), right flank (lateral), pubic bone and the inguinal ligament (inferior). This provided an excellent reconstitution of the patient’s abdominal wall. The two large skin and soft tissue flaps were then closed (Figure 5). This left a three-cm central area to heal by negative suction therapy and secondary intention.

**Figure 6:**
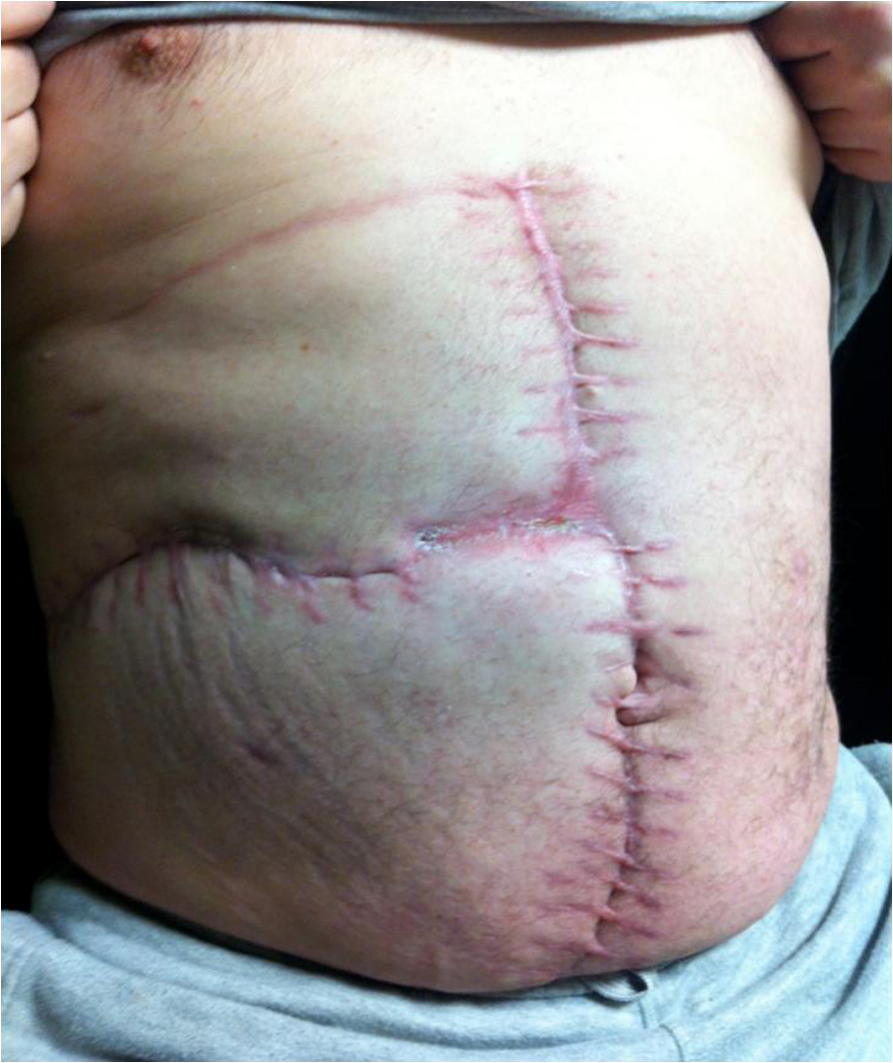
Abdominal wall 12 months after the reconstruction.

This patient was discharged home 34 days after his initial excisional biopsy with no evidence of organ failure. His 12-month out-patient follow-up continues to show no obvious hernia or attenuation in his abdominal wall (Figure 6).

## Discussion

Although up to 80% of necrotizing soft tissue infections are polymicrobial, *Clostridium* species are still associated with the classic presentation of gas gangrene myonecrosis [3]. Typically the presence of soft tissue gas, as observed in this patient, offers a grave prognosis [4]. In addition, gas gangrene of the torso carries an even worse outcome given the typical requirement for radical full thickness debridement that often results in open abdominal and thoracic cavities with exposed viscera [2]. Because of the tremendous risks associated with soft tissue coverage and eventual reconstruction, our patient underwent an alternative strategy. By maintaining two large, well-vascularized subcutaneous flaps as potential coverage, in addition to the internal oblique aponeurosis as a barrier to the peritoneal cavity, we were able to maintain significant reconstructive options. Given the risk inherent in this initial approach, the patient was returned to the operating room for a second tissue evaluation only eight hours after the initial debridement. As a consequence of his radical improvement in physiology and tissue quality, we persisted with this methodology.

In a significant number of patients (40%), a source of the necrotizing soft tissue infections is not readily identifiable [5]. In our patient, the initial lymph node biopsy was clearly the index insult; however, the specific source of the *Clostridium* is unknown. The gastrointestinal tract was not injured and this species is atypical for our hospital. We postulate that the preoperative skin preparation was insufficient to remove all the patient’s topical bacteria given his employment as a sewer plumber. This is particularly plausible given the known ability of *C. perfringens* to remain quiescent in tissues and then initiate a clinical infection when minor trauma (lymph node biopsy) provides an opportunity for growth (diabetes mellitus) [2]. We also believe the lack of skin changes and crepitus in the initial phases were a result of the patient’s general obesity. Furthermore, it is clear that the immunosuppression associated with his diabetes mellitus represented a significant co-factor in the rapid progression of this disease [2,6]. In addition, his final confirmed diagnosis of follicular lymphoma may also have negatively impacted his immune status [7].

Although our avoidance of a complete resection of all layers of the patient’s abdominal wall represents a significant departure from traditional dogma, the principles requiring a normal-appearing margin of tissue were maintained. Although the use of biologic materials represents a significant advancement in the field of surgery, this risk-adjusted technique of partial debridement has been echoed by previous authors in the context of clostridial myonecrosis [8,9]. Despite this technique, the risk of a subsequent massive ipsilateral abdominal wall hernia was substantial. As a result, we felt reconstruction of the abdominal wall with a prosthesis was essential. Given the reported improvements in function and durability of biologic materials in infected fields, two large pieces were inserted [10]. There is no current evidence on which to base the performance of biologics in the setting of *C. perfringens*. At a 12-month follow-up however, the patient’s abdominal wall remains well healed, with no drainage and no torso asymmetry. This is particularly important inferior to the arcuate line given the naturally thin posterior rectus sheath and aponeurosis components.

## Conclusion

The management of this patient represents a significant departure from the classic concepts surrounding clostridial myonecrosis. Although basic principles associated with radical debridement were maintained, a full thickness torso wall resection was avoided. This provided reconstruction advantages that included endogenous subcutaneous flap coverage, separation of the peritoneal cavity by the internal oblique aponeurosis, and prevention of a subsequent hernia below the arcuate line. These principles must be individualized to any specific patient.

## Consent

Written informed consent was obtained from the patient for publication of this case report and accompanying images. A copy of the written consent is available for review by the Editor-in-Chief of this journal.

## Competing interests

All authors declare that they have no competing interest.

## Authors’ contributions

CGB, JFO and IBA analyzed and interpreted all patient data. CGB, JFO, IBA, and AWK each made substantial contributions to writing of the entire manuscript. All authors read and approved the final manuscript.
